# Exploring anxiety awareness during academic science examinations

**DOI:** 10.1371/journal.pone.0261167

**Published:** 2021-12-15

**Authors:** Hippokratis Apostolidis, Thrasyvoulos Tsiatsos

**Affiliations:** Software and Interactive Technologies Laboratory, School of Informatics, Aristotle University of Thessaloniki, Thessaloniki, Greece; Sheffield Hallam University, UNITED KINGDOM

## Abstract

There is a developing interdisciplinary research field which has been trying to integrate results and expertise from various scientific areas, such as affective computing, pedagogical methodology and psychological appraisal theories, into learning environments. Moreover, anxiety recognition and regulation has attracted the interest of researchers as an important factor in the implementation of advanced learning environments. The present article explores the test anxiety and stress awareness of university students who are attending a science course during examinations. Real-time anxiety awareness as provided by biofeedback during science exams in an academic environment is shown to have a positive effect on the anxiety students experience and on their self-efficacy regarding examinations. Furthermore, the relevant research identifies a significant relationship between the students’ anxiety level and their performance. Finally, the current study indicates that the students’ anxiety awareness as provided by biofeedback is related to their performance, a relationship that is mediated and explained by the students’ anxiety.

## Introduction

The successful detection of emotions is a complicated procedure, which encompasses many relevant issues. One such issue is the interpretation of the emotional state changes that occur during a test activity, after taking into account all possible influencing factors [1].

An interesting study on undergraduate students involving biofeedback sessions showed a reduction of anxiety levels and an improved academic performance. In these sessions, the students participated in training activities, such as deep breathing and more, which aimed to provide relaxation [2]. However, Aritzeta, Soroa, Balluerka, Muela, Corostiaga, and Aliri [2] state that the current research on the topic of biofeedback in relation to university students is very limited and without established results. This suggestion motivated the current study, which focuses on student anxiety awareness by utilizing a biofeedback system applied during university examinations.

Zeidner [3] notes that test anxiety consists of more than one component, thus implying that it is more important to examine each individual component and how it relates to performance, rather than the relationship of performance evaluation to test anxiety as a whole. Liebert and Morris [4] suggest that test anxiety consists of two basic dimensions: (a) the cognitive dimension labelled as “worry”, which refers to concerns about being evaluated and to possibilities of failure and (b) the affective dimension labelled as “emotionality”, which refers to the perception of the autonomic reactions evoked by test conditions [5]. Moreover, Knox, Schacht and Turner [6] state that performance anxiety and test content are both components of test anxiety. They also note that “failure to manage test anxiety can result in failing courses, dropping out of school, a negative self-concept and a low earning potential”. Thus, it is very important for the students themselves to recognize their anxiety levels when taking part in difficult and challenging educational activities, in order to apply self-regulation strategies.

Our study investigates the emotionality component of test anxiety in relation to science courses and, more specifically, to a tertiary level computer science course. It focuses on providing biofeedback after measuring the users’ arousal–anxiety levels during examinations related to such a course (i.e. computer science). The present study refers to anxiety measurements which can be expressed more accurately as the feedback obtained with regard to the physiological arousal level estimations provided by the utilized biofeedback system. It is a preliminary attempt to support forms of emotion regulation, such as the students’ control of the anxiety they experience and of their self-efficacy during exams. Anxiety awareness is expressed through biofeedback information, which is revealed by using human physiological measurements. The information pertains to the following parameters: (a) galvanic skin response (GSR), (b) skin temperature (SKT) and (c) heart rate (HR). Moreover, the study deals with the use of the diaphragmatic breathing technique to support the self-regulation of anxiety. This well-known technique was chosen among others because it can be easily applied in almost all cases.

The present paper is structured as follows: Section 2 refers to related works. Next, the research questions of the study are introduced. The fourth section presents the materials used, the participants, the instruments and the methodology followed in order to conduct our research. This section also presents an evaluation activity using real-time biofeedback measurements. The evaluation results are then presented and discussed in the fifth section. The final section summarizes the concluding remarks and outlines our future steps.

## Related work on test-related anxiety observations

### Test anxiety

Many studies have demonstrated that the high test anxiety levels felt by students have a considerable effect on bad test performance [7–10]. Bettina Seipp [11] states that the two recognized components of test anxiety, i.e. worry and emotionality, are not examined separately in most studies, since “they are included intuitively in nearly all the questionnaires”. The present study explores the emotionality dimension of university students’ test anxiety. It suggests that, when this component exceeds an optimum level, then the students’ performance gradually declines during examinations.

The restriction of working memory capacity could be considered a factor that indicates whether one’s anxiety level has increased to such an extent that their performance is influenced or not [12]. However, this suggestion requires further research. In the case of our research, we shall try to explore similar critical factors through an interaction between biofeedback, test anxiety and performance.

A recent review of strategies dealing with high anxiety in educational contexts has revealed that the most effective way to handle stressful conditions is through a behavioral intervention that focuses on the students’ emotional states and is provided by biofeedback [2,13]. However, studies exploring the effect of biofeedback on university students are very limited and their results are debatable [2]. For example, Whited, Larkin and Whited [14] observe no clear anxiety reduction, when applying a biofeedback technique based on heart rate variability (HRV). This result is in contrast to other studies claiming that biofeedback has a positive effect both on general anxiety and on test anxiety specifically [2,15].

### Self-efficacy

Bandura [16,17] proposes a cognitive construct, which he names self-efficacy, that is related to the psychological procedures governing people’s beliefs about their capabilities. Scientific research reveals that low levels of self-efficacy are related to high anxiety [18,19]. Emotional inefficacy and high anxiety cause deficient self-regulation [20,21] and low academic efficacy, which is one of the main sub-scales of self-efficacy that is strongly related to academic achievement [22]. Zohar [23] notes that self-efficacy is a significant predictor of test performance achievement. He claims that self-efficacy has an indirect effect on test performance mediated by test anxiety, although there is the possibility of a direct effect between self-efficacy and achievement. Emotional arousal is the main source through which biofeedback functions. People are more likely to succeed when they are relaxed than when they are physiologically aroused.

Therefore, when biofeedback manages to reduce anxiety, one’s self-efficacy increases. This behavioral process leads to a higher possibility of improved academic performance. Furthermore, this process could imply that in many cases state anxiety has an inverse relationship with the self-efficacy felt at the moment the students’ anxiety increases or decreases. Further research is however required to clarify the relationship between state anxiety and self-efficacy. Scientific articles argue that there are two types of self-efficacy, general and task-specific. General self-efficacy represents an individual’s self-belief that s/he can complete any task at any time. Task-specific efficacy involves an individual’s self-belief that s/he can manage a specific function [24]. Bandura [16,17] claims that self-efficacy should be conceptualized in a condition-specific manner. However, it is commonly believed that general self-efficacy deals with a broader range of human behaviors. In addition, we maintain that people with high levels of general self-efficacy cope with affective processes in a better way [17]. Several articles show that there is a significant negative correlation between self-efficacy and anxiety towards examinations among students [25–27]. Thus, in the present research we address general self-efficacy as a more descriptive factor of the coping outcomes delivered through the benefits of biofeedback.

### Academic performance

New areas of educational technology research are now investigating affective learning environments. These environments can be viewed as a combination of learning environments and affective computing applications. Landowska [28] claims that there are two kinds of affective learning environments, depending on their human-computer interaction and communication level: (a) the one-way and (b) the two-way learning environment. One-way communication occurs when feedback observations or simulations take place but there is no system reaction. Such systems are called affect monitors. These systems, applied during the learning process, provide the students’ emotion measurements, as part of a larger learning analytics set of various measurements [29]. Two-way affective communication occurs when affective information is followed by a related reaction and there is a cycle of affective communication in human-system interactions [28].

Although educational technology is evolving and is being applied in affective learning environments, research on the impact of test anxiety on academic performance, especially in the case of university students, is still limited [2] and is mainly supported by relevant pre- & post-examination questionnaires. Mallow [30] uses the term ‘science anxiety’ when describing the anxiety noted in the case of educational activities and examinations related to science. Several studies have been carried out involving students at Loyola University Science Anxiety Clinic [31–33] to assess its effect, which have indicated that ‘science anxiety’ gravely impedes academic learning and performance. However, research on science anxiety is still limited [31]. Furthermore, as far as we know, there are no studies to date on science test anxiety awareness as provided by biofeedback and its effectiveness.

The present research is an attempt to combine the use of pre- and post-examination questionnaires with real-time physiological measurements to reveal some indications of the relationship between test anxiety, its awareness as provided by biofeedback and academic performance in relation to science courses. This is a preliminary attempt to explore real-time science anxiety awareness during examinations following one-way communication. It uses more than one physiological technique that is highly correlated to human anxiety levels [34] to detect some of the most common physiological reactions, which establish multiple bio-signal stress identification [35].

Based on the relevant literature, the GSR signal is a biomarker of sympathetic nervous system activation [36–38] and is considered one of the most critical markers describing emotional arousal and stress. In addition, the SKT signal has been used in multiple studies for emotion and stress detection [36,39], while the HR signal is considered to be closely correlated to stress [40,41]. The EEG signal is decomposed into five different sub-bands, namely delta (1-4Hz), theta ($-8 Hz), alpha (8-13Hz), beta (13-32Hz) and gamma (>32Hz), with rapid beta wave frequencies and fluctuating asymmetry characterized as the main stress indicators [42]. According to several studies, the most common physiological measures of stress detection include electroencephalography (EEG), heart rate (HR) and galvanic skin response [43–47]. Moreover, the analysis of biofeedback information combined with academic performance could potentially highlight new opportunities and practices regarding psychological and pedagogical interventions that focus on students’ affective regulation in combination with their academic goals and coping strategies.

## Research questions

As mentioned above, the present study is an attempt to investigate the science test anxiety felt by students. The main issues to explore include: (a) the effect of the students’ anxiety on their performance; and (b) the impact of their anxiety awareness as provided by biofeedback on their mentality in relation to academic achievement. To this aim, the research questions in this paper are the following:

- RQ1: Does the anxiety level information provided by biofeedback to students influence the anxiety they experience before a science examination?
- RQ2: Does the anxiety level information provided by biofeedback to students influence their self-efficacy during a science examination?
- RQ3: Is there any significant relationship between a student’s test anxiety and his/her performance?
- RQ4: How is the students’ anxiety awareness as provided by biofeedback related to their examination performance?

## Method

### Participants

The study involved 40 postgraduate students (16 males and 24 females, M = 26.31 y.o., SD = 2.11) who were attending a course in the field of computer science. Before the assessment activity, all participants read and signed a copy of written consent form, describing in detail the activity procedure, the purpose and the benefits of the research. Then, they completed an online biographical data questionnaire, which included questions regarding the students’ age and frequency of computer use. The results showed that all the participants used computers on a regular basis. Apart from science course attendance and frequency of computer use, no other inclusion or exclusion criteria were applied during the participants’ recruitment. As far as age and field of study are concerned, the sample can be considered representative of students taking exams for a bachelor’s or master’s degree in science. The study was conducted in accordance with the Declaration of Helsinki, and the protocol was approved by the Ethics Committee of the Aristotle University of Thessaloniki. It should be noted that this research is part of a PhD and that the present methodology has been reviewed by the competent research committee.

The results indicated that the students used computers on a regular basis.

### Instruments

#### State anxiety inventory

It involves the state part of the State-Trait Anxiety Inventory (STAI) [48], which consists of 20 items. The term “state anxiety” refers to an assessment of the intensity of the anxiety felt at any given time. Thus, in this case, the said instrument evaluates the intensity of the anxiety a student feels as s/he completes the questionnaire. Prior to the examination activity, the psychologist conducted an oral interview with each participant. The interview questions were based on the state part of the Greek adaptation of the STAI inventory, with a Cronbach’s alpha internal consistency of .93 [49]. Each item-question was rated on a 4-point Likert scale (1 = not at all, 2 = somewhat, 3 = moderately so, 4 = very much so). The STAI result-score was the sum of the scores assigned to each item. Some of the items were reverse-scored when the absence of anxiety was implied.

#### Knowledge test

The data collection method used for the assessment of exam performance was a knowledge test constructed by the course professor. The test included multiple-choice questions relevant to the syllabus of the course in relation to which the experiment took place. The test questions assessed basic factual knowledge of multimedia systems. The questions were shuffled and the test was divided into two equivalent parts. The total number of test items was 40 and, therefore, each of the two parts contained 20 questions.

One part was used during the first session of the study, whereas the other was used during the second session. The maximum total score for the examination was 20, which means that each correct answer was awarded 0.5 points. The reliability of the knowledge test was estimated by using the Kuder-Richardson [50] Formula 20 (KR-20) measure that checks the internal consistency of measurements with dichotomous data. The first part of the test had a KR-20 score of .726, whereas the score for the second part was .769. The overall KR-20 score for the knowledge test was .863.

The construct validity of the knowledge test was confirmed by the instructor of the course, who is an expert in the domain of multimedia systems.

#### The PHCC test anxiety questionnaire

The PHCC (Pasco Hernando Community College) test anxiety questionnaire is an instrument used to determine the anxiety students experience before an examination [51]. It consists of 10 (ten) items. Each item (i.e. question) is scored on a 5-point Likert scale, namely 1 = never, 2 = rarely, 3 = sometimes, 4 = often and 5 = always [52].According to the authors of the PHCC test, if the sum of the response scores is higher than 35, the anxiety level of the subject is unhealthy and s/he must apply certain strategies to reduce his/her anxiety. However, if the sum of the response scores is close to 10, then the subject is slightly too relaxed and in a context where a little more anxiety is necessary [51].

#### The GSE questionnaire

It is a questionnaire related to the participants’ self-efficacy, known as the “General Self-Efficacy” (GSE) scale [53,54]. According to its authors, the questionnaire assesses the general self-belief that one can handle difficult situations. It consists of ten items. Each item is scored on a 4-point Likert scale (1 = not at all true, 2 = hardly true, 3 = moderately true and 4 = exactly true). The total score of this assessment is the sum of all items. The score ranges from 10 to 40 and a higher score correspondingly indicates higher self-efficacy.

#### Anxiety awareness

**Fig 1:**
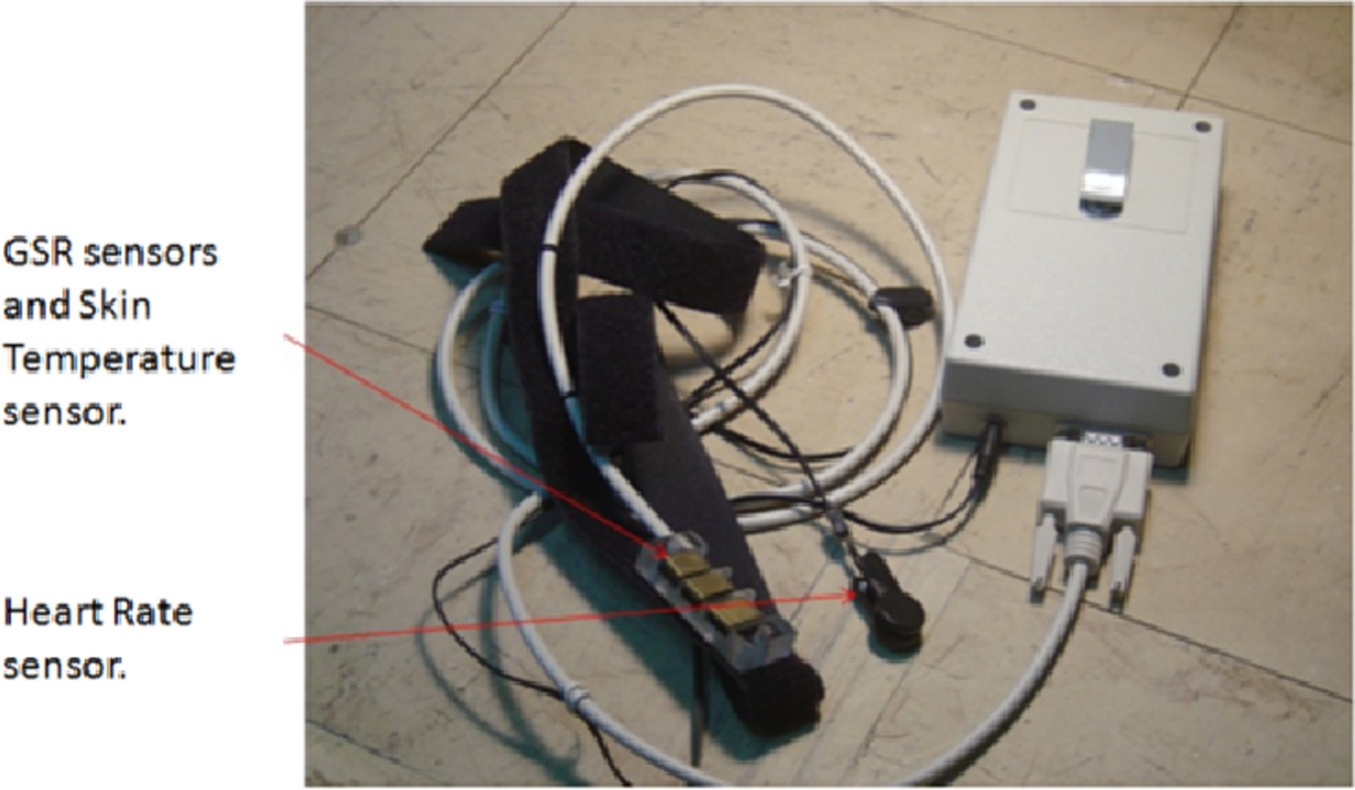
Biofeedback device.

The students’ science test anxiety during the examination was measured using a biofeedback device (Fig 1) [55] that can detect bio-signal measurements and classify these measurements into anxiety levels. The device collects three types of human bio-signals, namely skin temperature, galvanic skin response and heart rate.

The bio-signal values are sent to a Java biofeedback application by using the open source Weka library [56], which is a collection of machine learning algorithms. More specifically, this application uses a trained Gaussian regression algorithm [57] to classify the user’s bio-signals into anxiety levels on the fly, with an average root-mean-square error of 6.89 (RMSE). The RMSE is a common regression metric, and a standard way of measuring the error in predicting quantitative data. This metric can be expressed as the distance (sum of squared differences) between the predicted and actual values of a regression model. Since an RMSE value of 0.0 is the perfect metric for a regression model, which means that the relevant prediction is 100% correct and contains no errors, we can assume that the lower the achieved RMSE value the better [58]. Thus, the derived RMSE value average for each participant’s model is quite satisfactory. The user’s anxiety level is characterized by a numerical value ranging from 0-total relaxation to 100-total anxiety. Every second, the value of each user’s anxiety level and his/her bio-signals are stored in a database.

The graphical user interface of the biofeedback application receives the result of the regression procedure and displays a visualized response on the computer screen, where the subject of measurement can recognize his/her anxiety states through a chromatic code (e.g. red for high anxiety, green for relaxation) found next to his/her anxiety level indication. Every time the user reaches high anxiety levels, the application encourages him/her to use diaphragmatic breathing. In the present case, the psychologist supporting the study advised the students to apply the diaphragmatic breathing technique three times, whenever required.

Next, a real-time communication task is performed within the biofeedback application, which sends each real-time user’s anxiety value to another central application via TCP; this involves a monitoring application that displays the users’ codes and the anxiety levels of all participants during an activity that takes place on-site or from a distance.

The biofeedback device is based on the open source electronics prototyping platform Arduino Duemilanove (http://www.arduino.cc). It can be connected to a personal computer via a USB cable or operate in wireless mode (Bluetooth). The resolution of the Arduino board is 4.9 mv. The heart rate is calculated using a grove ear clip kit with a measurement range ≥ 30/min. A TMP36 temperature sensor that operates under a broad range of environmental conditions (-40° to 150°C) is also used. The said sensor has a ±2°C accuracy over temperature and a scale factor of 10 mV/ °C. The device-sampling rate is 10 Hz. Special care was taken so that the anxiety measurement using the biofeedback device took place at a normal environmental temperature in the range of 22°C to 24°C.

### Procedure

The procedure consisted of three main steps: (a) prior to the students’ examination activity, (b) during the students’ examination activity and (c) after the students’ examination activity.

**Fig 2:**
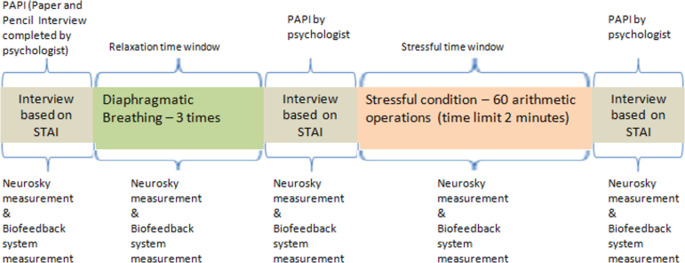
Psychometric test supervised by the psychologist.

*Step 1 –Prior to the examination activity*: According to Johnson [59], all people have different areas of emotional and intellectual sensitivity when trying to interpret and respond to current situations based on their experience. Thus, it is assumed that every participant is a unique human being and personality with their own specific bio-signal measurements. For this reason, it was decided that a psychometric test, supervised by the psychologist who supported this research, should precede the scheduled examination. The psychometric test included an interview process in the form of a paper and pencil interview (PAPI) that was completed by the psychologist, following the protocol described in Fig 2. The interview questions were based on the state part of the State-Trait Anxiety Inventory (STAI) [48] that was translated into Greek. The psychologist had the opportunity to discuss all interview questions with each participant and provide support so that they could clearly express their emotional state at the time. During this psychometric test, the subject was simultaneously measured using two bio-signal devices: (a) the biofeedback device [55] and (b) the mindwave mobile headset by Neurosky Company (http://www.neurosky.com), which is a commercial electroencephalogram (EEG) biosensor solution. The Neurosky device was used to calibrate the biofeedback measurements. During the psychometric test, the psychologist tried to make the students feel emotions which would simulate two affective states, anxiety and relaxation. Thus, each participant was asked to apply the diaphragmatic breathing technique three times in order to feel relaxed. Then, at a next stage, each student was asked to answer a form with sixty (60) arithmetic operations in two (2) minutes in order to experience a certain level of anxiety. This task forms part of the cognitive ability/intelligence tasks battery. It is called the Number Facility test (NF) and taps arithmetic operation fluency [60].

The biofeedback device anxiety level values have a range similar to the Neurosky anxiety measurements. The range is from 0 to 100. The anxiety level provided by biofeedback decreases when the user is relaxed and increases when the user is stressed. Thus, an anxiety level between 1 and 20 is viewed as relaxation, 21–40 as limited relaxation, 41–60 as neutral, 61–80 as moderate anxiety and 81–100 as high anxiety. At the end of each phase of the psychometric test (relaxation or stressful condition), the psychologist made a rough comparison between the Neurosky measured anxiety and the interview results. The psychologist noticed that the interview results were consistent with the Neurosky measurements, in relation to whether the student was seen to be relaxed or to be getting anxious.

The anxiety values received from the Neurosky mindset class (Mindset library) [61] were used to create labels for the bio-signal measurements of the biofeedback device. The mindwave headset averaged a quality signal ranging from 80% to 100%. Each student’s anxiety reaction was initially indicated by the mindwave and was assigned to the biofeedback device measurements as an anxiety level (label). Each set of this labelled data formed a specific training set for each subject of measurement and was stored in a database. A supervised machine learning method was applied to each training set of labelled measurements in the form of <GSR, HR, SKT, Label> and the data was split into classes according to the labels. Each participant and his/her corresponding data set produced the participant’s trained model. This model was then used during the main activity to predict the class of the participant’s real-time bio-signals. After the psychometric measurement, the psychologist introduced coping and constructive emotion regulation strategies to the students, specifically linked to cognitive reappraisal. For example, the psychologist tried to persuade the students to avoid putting the blame on other reasons in case of bad academic performance and apply self-criticism instead. There are however many disputes in relation to this coping strategy. For example, Porter and Stone [62,63] have stated that it is mostly men who adopt a more direct attitude such as self-criticism under stressful conditions, whereas most women opt for avoidance. The psychologist introduced the “learning from our mistakes” strategy in an effort to help students avoid frustration in case of failure and guide them towards more positive and creative behavior [64–66], which could potentially improve their academic performance and decrease their anxiety vis-à-vis examinations. In addition, the psychologist took into account the relevant research which shows that self-criticism as a coping strategy is very closely related to quality of life [62,67], and that there is a significant connection between quality of life and stress [68,69]. The subjects then practised diaphragmatic breathing under the psychologist’s guidance. Before the whole examination procedure took place, the participants responded to two questionnaires on the anxiety students experience before an examination (PHCC) and on self-efficacy (GSE scale).

**Fig 3:**
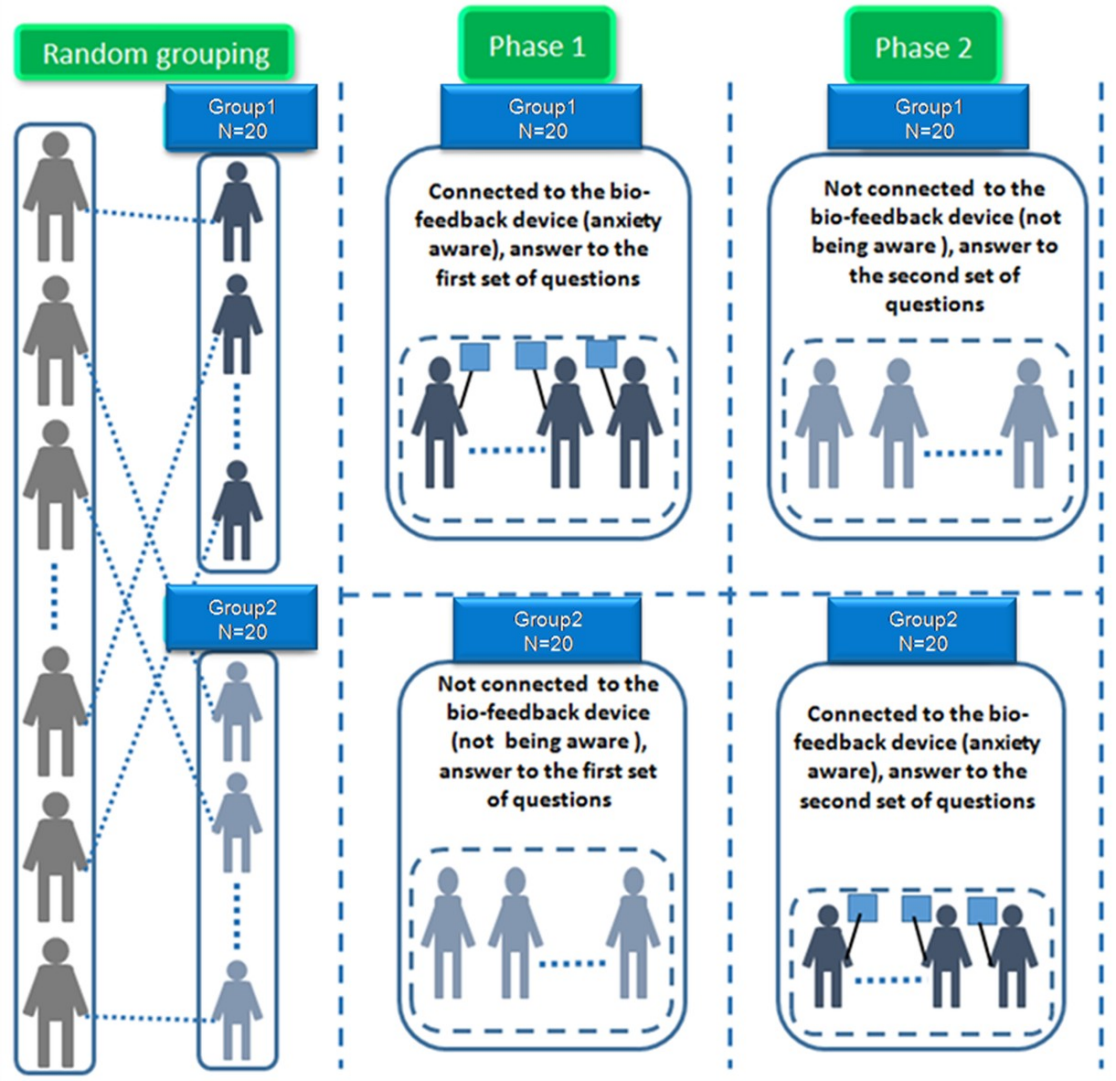
The activity procedure (student groups and phases).

*Step 2—During the examination activity*: The subjects’ examination was in the form of multiple-choice tests and was divided into two phases. The students answered the Knowledge Test presented in paragraph 4.2.2. They were randomly divided into two groups of twenty (20) students. The two phases of the examination (Fig 3) were as follows:

- Phase 1: In the first phase, the members of the first group (Group1) were connected to the measuring biofeedback devices and watched their biofeedback measurements while answering the first part of the Knowledge Test. The members of the second group (Group 2) were not connected to biofeedback devices. Thus, the members of the second group were not aware of their biofeedback measurements while answering the first set of questions in the examination.
- Phase 2: In the second phase, the members of the first group (Group1) were not connected to biofeedback devices. Thus, this group was not involved in any biofeedback intervention while answering the second part of the Knowledge Test. Instead, the members of the second group (Group2) were connected to the measuring biofeedback devices and watched their biofeedback measurements while answering the second set of questions in the examination.

*Step 3—After the portion of the examination activity that involved biofeedback*: The students responded to the questionnaires on the anxiety students experience during an examination (PHCC) and on self-efficacy (GSE scale).

PHCC and GSE questionnaires were handed to the students before the whole activity took place and after the part of the examination that involved biofeedback, in order to examine whether the biofeedback measurements supported the students by lowering their level of test anxiety and increasing their self-efficacy.

When the students were connected to the biofeedback device, they could monitor their real-time anxiety levels through a biofeedback application running on their computer. The graphical part of the biofeedback application displayed the real-time predicted anxiety levels as a visualized response, a description of their current anxiety state, and a chromatic code (red for anxiety, orange for moderate anxiety and green for relaxation). In case of high anxiety, a tip appeared on the computer screen encouraging the participants to use diaphragmatic breathing. Meanwhile, the instructor also had access to the monitoring application that was keeping track of all anxiety measurements. All phases of the activity were synchronous and took place in the classroom. The whole examination involved a specific type of web-based online multiple-choice questions. The performance results and time period of each student’s answers were stored in a database. Moreover, every biofeedback measurement was also stored in the database, along with its timestamp.

### Data analysis

Within-subjects data analysis, which is based on the PHCC test anxiety and GSE scale questionnaires, was used to examine whether there was a significant difference between the pre- & post-activity responses.

The dependent sample t-test was applied to the PHCC responses (pre and post activity) that examined the first research question: “Does the anxiety level information provided by biofeedback to students influence the anxiety they experience towards a science examination?” In addition, the dependent sample t-test was applied to the GSE responses (pre and post activity) that examined the second research question: “Does the anxiety level information provided by biofeedback to students influence their self-efficacy during a science examination?”

Cronbach’s alpha coefficients were examined for each questionnaire in order to test their internal consistency. According to Nunnally [70], alphas above .70 are acceptable. A bivariate analysis was then applied to examine the third research question: “Is there any significant relationship between a student’s test anxiety and his/her performance?” This correlation analysis measured the strength of the relationship between each student’s right or wrong answers at specific timestamps, and his/her corresponding anxiety levels at the same timestamps.

Finally, a mediation analysis was used to explore the fourth research question: “How is the students’ anxiety awareness as provided by biofeedback related to their examination performance?”

SPSS version 21 was used for the statistical analysis and the evaluation results are presented in the section below.

## Evaluation results and discussion

### Student anxiety towards science examinations

The Cronbach’s alpha consistency estimates obtained from the PHCC questionnaire when completed prior to and after the examination activity were .86 and .92 respectively. In order to examine the first research question (RQ1), we decided to apply the paired samples t-test in order to observe whether the students’ PHCC scores differed, based on whether they completed the questionnaire before or after the relevant activity. Since the sample was lower than 50 (N< = 50), we used the Shapiro-Wilk test to examine whether the two variables of the PHCC scores (pre- and post-activity responses) were well modelled and followed a normal distribution. The result was p < .05 for both data sets, which indicated that the two variables did not follow a normal distribution [71].

Since the two data sets of PHCC scores did not follow a normal distribution, we applied the non-parametric Wilcoxon (paired samples) test. The significance obtained was p = .01 < .05, which indicated that the distribution of the PHCC scores was significantly different across the two categories, due to the completion of the questionnaires before and after the activity. Since this case is a non-parametric Wilcoxon test, two formulae commonly used under relevant conditions were used to calculate the effect size.

The first one suggested by Field [72] uses the equation $r=\frac{z}{\sqrt{n}}$ [73], where z is the standardized test statistic derived from the Wilcoxon signed-rank test and n is the sample size, resulting in an effect size of-.40 (z = -2.56, n = 40, S6 Table). This effect size (cardinal number) belongs to the r family (which measures the strength of association) and, considering that a correlation r of .5 indicates 25% of the variance explained by the difference between the groups, means that the resulting value could be scaled as a medium effect size [73,74]. The application of the pwr library in R language (pwr.t.test) with a sample size of 40 and the aforementioned effect size result in a power calculation of .69.

The second formula is Cliff’s Delta and is recommended in cases of non-parametric effect size measurement for two groups of categories beyond the p values’ interpretation [75]. The present study used Python programming language based on Torciano’s [76] and Ernst’s [77] open source code repositories in order to apply Cliff’s Delta formula. The resulting effect size was .31 which, according to Cliff’s Delta scale criteria (.147 = small, .33 = medium, .47 = large), is close to medium. In this case, the derived power calculation was equal to .46.

**Fig 4:**
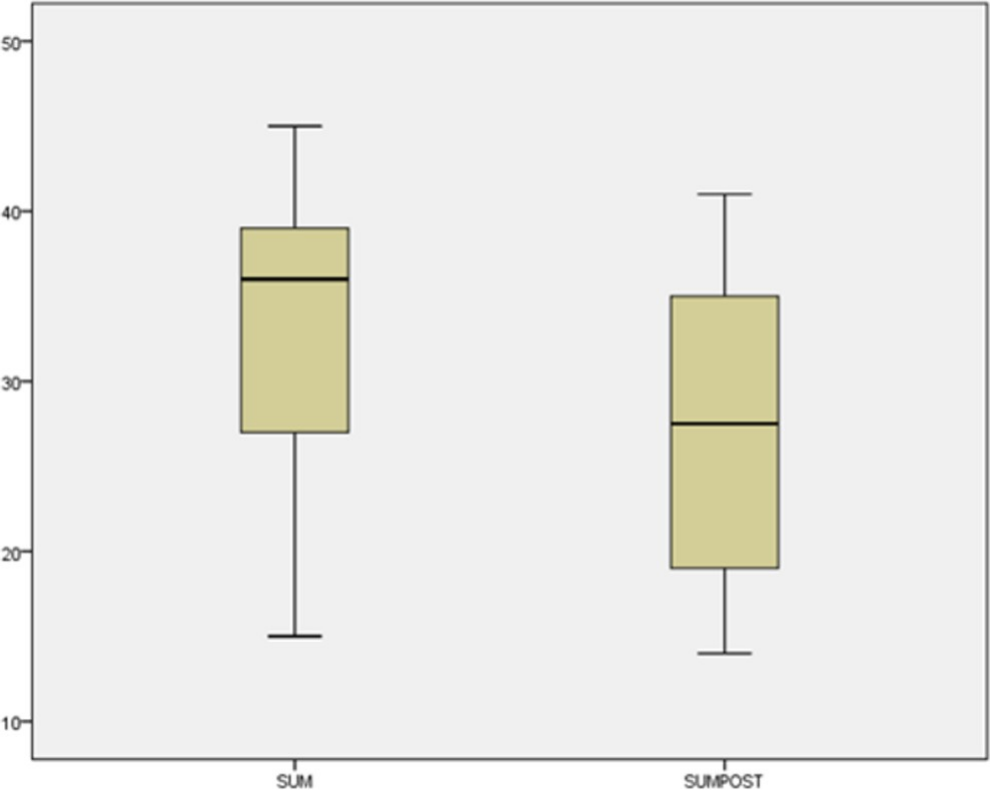
Box plot of PHCC scores (pre-activity SUM and post-activity responses SUMPOST).

Thus, we can conclude that both applied formulae resulted in a medium effect size and that there is a significant difference between the pre-activity and post-activity results related to the anxiety students experience when faced with examinations. The inspection of the box plot (depicted in Fig 4) reveals that the post-activity anxiety students experienced regarding the examinations (SUMPOST) was lower than their pre-activity anxiety (SUM).

### Student self-efficacy

The Cronbach’s alpha consistency estimates obtained from the GSE questionnaire when completed before and after the examination activity were .76 and .77 respectively. The resulting alphas are in line with the statements of the authors of the GSE questionnaire who refer to an internal reliability between .76 and .90 [53,54].

In examining the second research question (RQ2), the paired samples t-test was used to explore the means of the two data sets formed by the students’ self-efficacy responses, the criterion being whether the questionnaire was completed before or after the relevant activity. As the sample size is lower than 50 (N< = 50), the Shapiro-Wilk test was used to examine whether the two self-efficacy score variables (pre- and post-activity responses) were well modelled and followed a normal distribution. The obtained result was p > .05 for both data sets (significances of .26 and .06 respectively), which indicated that the two variables followed a normal distribution [71]. In addition, the paired sample correlation of the two variables (pre- and post-activity self-efficacy scores) is .633 > .4, p < .01, which shows that both variables are significantly positively correlated. Thus, we can explore the paired samples t-test further. The results received from the paired samples t-test are: (a) significance p = .00< .05 (95% confidence interval); (b) t-value with degrees of freedom t (39) = -3.80; and (c) effect size r = .52, which, according to Field [72], is slightly higher than the large effect size (.5). We applied the pwr library in R language for a sample size of 40 and the aforementioned effect size resulted in a power calculation of .67.

According to the above, we can conclude that there is a significant difference between the pre-test (Μ = 27.88, SE = .53) and the post-test self-efficacy of the students (M = 29.45, SE = .39). Furthermore, the t-value is negative, which signifies that the pre-activity self-efficacy mean is lower than the post-activity one [72].

### Relationship between anxiety level and student performance

When examining the third research question (RQ3), we applied a statistical analysis related to each student’s anxiety level during a specific time window (during his/her examination, when s/he was connected to the biofeedback device) and their related performance (0 = wrong answer, 1 = correct answer) at the same time.

More specifically, we tried to explore the statistical measure of the strength of the relationship between the two variables of anxiety level and performance for each subject of measurement.

In cases of normal distribution without outliers, we applied the point-biserial as a special case of Pearson’s product-moment correlation, which is recommended when one of the examined variables has a dichotomous outcome. In most of the examined cases however, outliers were present or the criteria of normal distribution were not satisfied, which meant that we applied Spearman’s rank-order correlation.

As an overall conclusion, we can observe that the majority of participants (28/40, 70%) presented a significant change in their performance related to (a) an anxiety level increment at higher values or (b) an anxiety level decrement at very low values, which could be characterized as a very relaxed area.

According to the evaluation results, three types of relationships have emerged between the students’ anxiety levels and performance. These are presented in the following paragraphs:

- *The students’ performance significantly deteriorates as their anxiety level increases*. From the statistical analysis, we noted that there was a significant negative correlation between anxiety and performance for 19 out of the 40 participants (47%). For example, when we examined the correlation between the anxiety level (LabelEmo variable) and performance (Perform variable) of the student with user-id 34, we obtained a Correlation Coefficient r = -.601**. Thus, we can conclude that there is a significant negative correlation between the students’ performance and their anxiety level increment.
- *The students’ performance significantly deteriorates when their anxiety level decreases (the users get too relaxed)*. From the applied statistical analysis, we noted that there was a significant positive correlation for 9 out of the 40 participants (23%). For example, when we examined the correlation between the anxiety level (LabelEmo variable) and performance (Perform variable) of the student with user-id 37, we obtained a Correlation Coefficient r = .634**. Thus, we can conclude that there is a significant positive relationship between the students’ performance and their anxiety level measurements.
- *There is no significant correlation between a student’s anxiety level and his/her performance*. Based on the collected data regarding a considerable portion of students (12/40, 30%), no correlation between their performance and their anxiety level was observed.

The significant correlation coefficients, both positive and negative, between the variables LabelEmo (students’ anxiety level) and Perform (students’ performance) for 28 out of the 40 students are presented in Table 1.

**Table 1:** Significant correlation coefficients between the students’ anxiety level and performance (for 28 students).

STUDENT	CORRELATION COEFFICIENT	CORRELATION	DISTRIBUTION	OUTLIERS
User 31	-.568**	Spearman’s	Normal	Outliers
User 34	-.601**	Spearman’s	Normal	Outliers
User 35	.569**	Spearman’s	Not normal	Outliers
User 37	.634**	Pearson	Normal	No outliers
User 38	-.653**	Pearson	Normal	No outliers
User 39	-.646**	Pearson	Normal	No outliers
User 40	-.602**	Spearman	Normal	Outliers
User 41	.500*	Spearman	Normal	Outliers
User 42	-.807**	Pearson	Normal	No outliers
User 43	-.791**	Pearson	Normal	No outliers
User 331	.721*	Spearman	Not normal	Outliers
User 332	-.773**	Pearson	Normal	No outliers
User 333	-.814**	Spearman	Not normal	Outliers
User 335	-.757**	Spearman	Not normal	Outliers
User 336	-.619**	Spearman	Not normal	Outliers
User 337	-.850**	Spearman	Not normal	No outliers
User 339	.806*	Spearman	Not normal	No outliers
User 340	-.798**	Spearman	Not normal	Outliers
User 341	-.780*	Spearman	Not normal	Outliers
User 342	-.761**	Spearman	Not normal	Outliers
User 344	.687**	Pearson	Normal	No outliers
User 347	.480*	Spearman	Not normal	Outliers
User 441	-.850**	Spearman	Not normal	Outliers
User 442	-.918**	Pearson	Normal	No outliers
User 444	.894**	Pearson	Normal	No outliers
User 447	-.947**	Pearson	Normal	No outliers
User 449	-.744*	Spearman	Not normal	Outliers
User 450	.795**	Spearman	Normal	Outliers

### The relationship between the anxiety awareness provided by biofeedback and the students’ performance

**Fig 5:**
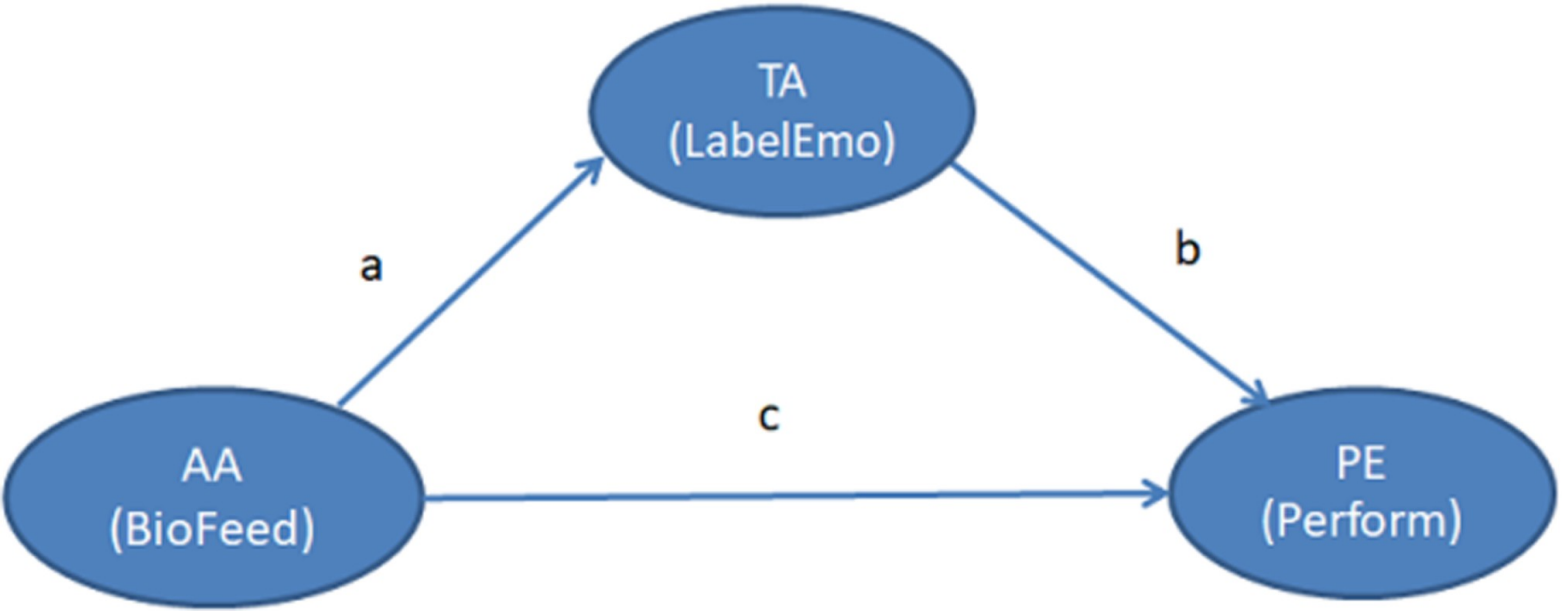
Test anxiety (TA) as an indirect effect between anxiety awareness as provided by biofeedback (AA) and performance (PE).

When examining the fourth research question (RQ4), we carried out a within-subject mediation analysis [73]. During the statistical analysis in question, we used test anxiety (TA) as the mediator (intervening variable) of the relationship between the predictor, namely the students’ biofeedback (biofeedback, AA), and the outcome, namely the students’ performance (PE). In Fig 5, the displayed mediational effect, whereby AA leads to PE through TA (i.e. paths a, b), is characterized as the indirect effect. The indirect effect represents the portion of the relationship between AA and PE that is mediated by TA [78].

First, we examined the confirmation of the above-mentioned indirect effect by applying the well-known “multiplication of two regression coefficients” technique [79]. The analysis showed that there is a significant indirect effect of anxiety awareness as provided by biofeedback on student performance, which is established by the results, i.e. Sobel test = -3.44 (< -1.96) and p < .001.

Next, we examined whether this indirect effect is a case of full or partial mediation. In the above-mentioned case, the mediator candidate and the outcome variables are dichotomous (values 0 and 1). Therefore, in order to examine the mediational effect of test anxiety, we proceeded in line with [80]:

1. The regression coefficient between anxiety awareness as provided by biofeedback and performance (path c, Fig 5) was statistically significant (p = .000) (Table 2). The independent variable of anxiety awareness as provided by biofeedback is shown to have a significant positive effect on performance.

**Table 2:** Linear regression between biofeedback and performance.

Coefficients^a^						
Model		Unstandardized Coefficients		Standardized Coefficients	t	Sig.
		B	Std. Error	Beta		
1	(Constant)	,358	,032		11,373	,000
	BioFeed	,322	,044	,322	7,360	,000

a. Dependent Variable: Perform.
2. The regression coefficient between biofeedback and test anxiety (path a, Fig 5) was statistically significant (p = .000) (Table 3). This indicates that the biofeedback has a significant negative effect on test anxiety.

**Table 3:** Linear regression between biofeedback and test anxiety.

Coefficients^a^						
Model		Unstandardized Coefficients		Standardized Coefficients	t	Sig.
		B	Std. Error	Beta		
1	(Constant)	60,457	1,268		47,691	,000
	BioFeed	-6,722	1,759	-,174	-3,821	,000

a. Dependent Variable: LabelEmo.
3. The regression coefficient between test anxiety and performance (path b, Fig 5) was statistically significant (p = .000) (Table 4). This finding shows that test anxiety has a significant negative effect on performance.

**Table 4:** Linear regression between test anxiety and performance.

Coefficients^a^						
Model		Unstandardized Coefficients		Standardized Coefficients	t	Sig.
		B	Std. Error	Beta		
1	(Constant)	,843	,070		12,006	,000
	LabelEmo	-,006	,001	-,215	-4,773	,000

a. Dependent Variable: Perform.
4. We performed a multiple regression analysis with biofeedback (BioFeed) and test anxiety (LabelEmo) predicting performance (Perform). The resulting regression coefficients were statistically significant (in both cases p = .000) (Table 5). This finding leads to the conclusion that there is a partial mediation of test anxiety between biofeedback and performance [80].The multiple regression coefficient of biofeedback predicting performance (d_BioFeed-LabelEmo-Perform_) in this (fourth) step is lower than the relevant simple regression coefficient in the first step (c_BioFeed-Perform_), which indicates that the test anxiety intervention decreases the effect of biofeedback on performance.

**Table 5:** Linear regression between biofeedback, test anxiety and performance.

Coefficients^a^						
Model		Unstandardized Coefficients		Standardized Coefficients	t	Sig.
		B	Std. Error	Beta		
1	(Constant)	,615	,075		8,180	,000
	LabelEmo	-,004	,001	-,164	-3,751	,000
	BioFeed	,293	,044	,294	6,696	,000

a. Dependent Variable: Perform.

Therefore, as an answer to the fourth research question, we could suggest that there is a negative indirect effect of test anxiety on the positive relationship between biofeedback and student performance. The statistical analysis indicated that this indirect effect is a case of partial mediation.

### Main findings

This article is an initial attempt to investigate the possible impact of science test anxiety awareness as provided by biofeedback on student self-efficacy and performance during academic examinations.

The data collected and analysed in the previous sections has produced a number of interesting results regarding all of the research questions raised, which are presented in the following paragraphs. The reason we decided to use another physiological signal, namely the EEG, measured by a commercial device like the Neurosky in real time during the psychometric test, in order to label the biofeedback device measurements (GSR, EKT and HR), is that recent physiological studies support and endorse real-time bio-signal recording without disturbing or interrupting the participant’s activity and experience [81]. The use of a questionnaire as a self-reporting tool for detecting an affective state like anxiety interrupts the flow of interaction and does not necessarily present the participant’s actual state. Furthermore, the use of questionnaires includes the risk of human error due to response bias or questions being misunderstood [81,82].

Nevertheless, our calibration procedure was supported by interviews with the psychologist that aimed to confirm the resulting emotional states and avoid any misunderstanding. Arsalan et al [43] have stated that the combination of three methods, e.g. EEG, GSR and photoplethysmography (PPG), which is a technique used to calculate one’s heart rate (HR), increases stress detection accuracy. It is for this reason that we decided on a combination of GSR, HR, SKT and EEG signals in order to train each subject’s classification model and on a combination of GSR, HR and SKT signals for predicting real-time anxiety levels.

RQ1: Does the anxiety level information provided by biofeedback to students influence the anxiety they experience towards a science examination? The statistical analysis showed that the response scores for the majority of participants before the activity were significantly higher than those obtained after the portion of the activity that involved biofeedback. Thus, the students’ biofeedback supplied by our research significantly affected the anxiety they experienced regarding the examination, by reducing it to a healthier level. This is in accordance with the findings of biological and psychological research, which suggest that the avoidance of inner awareness may lead to a poor reaction under stressful situations [83].

RQ2: Does the anxiety level information provided by biofeedback to students influence their self-efficacy during a science examination? The statistical analysis indicated that the students’ self-efficacy significantly improved after the educational activity that involved biofeedback. This finding is in line with Schutz and Davis [84], who claim that during test taking “being in tune with our emotions can be very helpful in the self-regulation process”. Based on the results of our study, we could suggest that being in tune with their anxiety can be a supportive factor for students with regard to their self-regulation processes, in the sense of controlling their anxiety and leading them to perceivably improved self-efficacy.

However, concerning the discussion of the findings related to questions RQ1 and RQ2, we could suggest that further research is required to establish more unambiguous claims. There is always the possibility that the pre-post changes in PHCC and GSE scores are affected by the fact that the examination has been completed.

RQ3: Is there any significant relationship between a student’s test anxiety and his/her performance? The third research question refers to the relationship between the students’ performance and their anxiety level. For the majority of students (28/40, 70%), there was a significant correlation between their performance and their anxiety level. In the case of 47% of the students, it is evident that as their anxiety level increased, their performance decreased. This result is in line with several other findings in the relevant literature [85–87]. Furthermore, in his research on science anxiety, Mallow [30,31] suggests that high levels of anxiety seriously impede student learning and performance both in science education and science examinations. Moreover, there was a portion of students (23%) whose anxiety level decreased to very low levels (it could be assumed that the relevant measurements approximated the relaxed area) and their performance also deteriorated. Thus, we could assume that when students get too relaxed, they are no longer strongly engaged in an activity and are prone to make mistakes. These indications are in line with literature findings that mention that anxiety is significantly related to academic performance under difficult circumstances [12,15]. Moreover, we could claim that all students have their own specific anxiety level (which could be termed as moderate anxiety) where they perform better. If their anxiety goes below or above this level, by showing lower or higher values respectively, then their performance worsens and they make more mistakes. This finding is in line with Salend [88], who claims that “appropriate levels of stress can enhance students’ memory, attention and motivation leading to improved test performance”.

Test anxiety is a multidimensional construct consisting of cognitive, affective and behavioral components. Each component represents a separate channel through which test anxiety may be expressed in relation to test-taking situations. Worry, self-preoccupation and cognitive interference compose the cognitive facet of test anxiety [89]. Cognitive interference refers to unwanted and disturbing thoughts. There is the hypothesis that intrusive thoughts occurring in academic environments are functions of test anxiety and that these thoughts can disrupt task performance in anxious students [90]. Furthermore, these thoughts can influence the emotionality dimension of test anxiety, and consequently further increase one’s anxiety levels. In addition, a student who is engaged in a task with external motivation (e.g. who feels obliged to get good grades or fears failure) is likely to feel stressed when trying to avoid a negative outcome in his/her performance or may be highly fearful of the forthcoming outcome and therefore actually achieve the opposite of what s/he initially intended [91].

RQ4: How is the students’ anxiety awareness as provided by biofeedback related to their examination performance? According to the evaluation results, test anxiety has an indirect effect and acts as a partial mediator in the positive relationship between biofeedback and performance.

As shown in the aforementioned paragraphs, the positive effect between biofeedback and performance can be explained through the support the former provides to students in relation to the perceived anxiety they experience during examinations and to their self-efficacy. The participants in the study were advised and trained by the psychologist to practice diaphragmatic breathing three times whenever required. The effectiveness of this technique in reducing anxiety levels has been recorded in many studies [2,92,93]. In addition, although the anxiety information provided by biofeedback is shown to reduce test anxiety, the negative effects of anxiety are still found to exist, either increased or decreased on a case-by-case basis. The evaluation findings show that test anxiety acts as a suppressor of the positive impact of biofeedback on student performance. Several articles deal with the relationship between anxiety awareness and performance in general. Recent reviews suggest that biofeedback programs (like emWave) can reduce test anxiety and increase academic performance in both children and adolescents [2,94]. Furthermore, the biofeedback technique applied in many levels of education has shown that it improves “emotional wellbeing” and academic performance [2,95]. However, contrary to the aforementioned articles, the results of a recent study have indicated that the link between the use of biofeedback and academic performance is not so obvious [2,14,96]. We assume that this contradiction is due to the fact that an increase in test anxiety can suppress the positive effect of biofeedback on performance. This observation leads us to assume that there is a specific anxiety level threshold. When one’s test anxiety is below this threshold, then biofeedback is positively correlated to performance, but when one’s test anxiety increases above this threshold, then biofeedback cannot significantly reduce it, and the positive effects of biofeedback on performance are very limited, zero or even negative. It is also very likely that this threshold differs from person to person.

## Conclusions

The present study uses real-time biofeedback to provide anxiety awareness and recommends diaphragmatic breathing as a useful practice for handling anxiety during science examinations. The research evaluation findings have shown that the recommended anxiety awareness procedures significantly improve the anxiety students experience towards written examinations in relation to a science course, and increase their self-efficacy. Moreover, the study reveals a significantly positive effect of anxiety awareness as provided by biofeedback on the academic performance of students. However, test anxiety was also shown to act as a partial mediator in the relationship between biofeedback and performance, which is a fact that suppresses the positive effect of this relationship. Thus, we can support that biofeedback and the test anxiety awareness it provides can contribute towards an improved academic performance to some extent, however this field requires further research based on experiments that should focus on the interaction between the biofeedback application and its users.

## Limitations

The present study suffers from certain limitations that should be taken into consideration when interpreting its findings. Firstly, the sample size of the experiment is relatively small, which means that more experiments should be performed. With a confidence level of 95% and a margin of error of 5% approximately, the required sample size should have included about 380–400 participants. Unfortunately, it is impossible to carry out the present study with such a large sample, as the number of biofeedback devices is limited. The sample size of 40 participants in our research resulted in a margin of error of 15.48% [97]. Secondly, the study assessed the impact of anxiety awareness on academic performance during examinations using a knowledge test that focused on the assessment of factual knowledge only.

Furthermore, a control—experimental group research design would have enabled an analysis of the relative change in anxiety levels due to the biofeedback condition, thus providing critical information and more concrete results.

## Future work

Further research is required in order to examine whether anxiety awareness is beneficial for assessing other types of knowledge, such as higher-level thinking skills. Moreover, the interaction between the students’ test anxiety, biofeedback and their working memory related to academic performance may prove to be an interesting area for future research work. In addition, an analysis that would include test anxiety and self-efficacy both as moderator or mediator variables of the relationship between anxiety awareness as provided by biofeedback and performance could potentially lead us to a better understanding of the relationships between biofeedback, test anxiety, self-efficacy and performance. A study of the aforementioned factors in conjunction with each subject’s personality traits may also reveal useful findings. The real-time anxiety awareness provided by biofeedback can contribute to a better understanding of behavioral changes over time which are closely related to performance. These thoughts could drive our future research with the ultimate goal to formulate an intervention framework that would support one’s motivation towards improved academic performance.

Furthermore, there is the intention to repeat these research experiments with more students, in relation to intensive autonomous or collaborative learning activities, in order to apply the biofeedback measurement technique in combination with the constructive reappraisal strategy and various pedagogical methodologies. More specifically, in the case of collaborative learning activities, there is the intention to use the students’ anxiety level as provided by biofeedback as a trigger to activate the appropriate adaptation pattern [98] in the form of an adaptive intervention tool.

Finally, more research is required in order to a) observe whether the students apply any introduced anxiety reducing intervention and b) divide the participants into two groups (control and experimental) and compare their respective PHCC and GSE questionnaire scores.

## Supporting information

S1 Table Frequency table for the pre-activity responses to the PHCC questionnaire. (DOCX)

S2 Table Frequency table for the post-activity responses to the PHCC questionnaire. (DOCX)

S3 Table Descriptive statistics for the pre and post-activity responses to the PHCC questionnaire. (DOCX)

S4 Table Test of normality of PHCC questionnaire responses. (DOCX)

S5 Table Wilcoxon signed ranks test of PHCC questionnaire responses. (DOCX)

S6 Table Wilcoxon signed ranks test statistics of PHCC questionnaire responses. (DOCX)

S7 Table Frequency table for the pre-activity responses to the GSE questionnaire. (DOCX)

S8 Table Frequency table for the post-activity responses to the GSE questionnaire. (DOCX)

S9 Table Test of normality between the pre- and post-activity responses to the GSE questionnaire. (DOCX)

S10 Table Paired samples correlations between the pre- and post-activity responses to the GSE questionnaire. (DOCX)

S11 Table Paired samples t-test between the pre- and post-activity responses to the GSE questionnaire. (DOCX)

S1 File Zip file containing data and output files from SPSS. (ZIP)
