# Supplementary material for: Exploring anxiety awareness during academic science examinations
Source: PLoS One. 2021 Dec 15;16(12):e0261167. doi: 10.1371/journal.pone.0261167 (PMC8673629; doi:10.1371/journal.pone.0261167)
Supplement: S1 Table — (DOCX) [file pone.0261167.s001.docx]

| **SUM** | | | | | |
| --- | --- | --- | --- | --- | --- |
|  | | Frequency | Percent | Valid Percent | Cumulative Percent |
| Valid | 15 | 1 | 2.5 | 2.5 | 2.5 |
|  | 17 | 1 | 2.5 | 2.5 | 5.0 |
|  | 25 | 4 | 10.0 | 10.0 | 15.0 |
|  | 27 | 2 | 5.0 | 5.0 | 20.0 |
|  | 28 | 3 | 7.5 | 7.5 | 27.5 |
|  | 29 | 2 | 5.0 | 5.0 | 32.5 |
|  | 34 | 1 | 2.5 | 2.5 | 35.0 |
|  | 36 | 5 | 12.5 | 12.5 | 47.5 |
|  | 37 | 4 | 10.0 | 10.0 | 57.5 |
|  | 38 | 6 | 15.0 | 15.0 | 72.5 |
|  | 39 | 4 | 10.0 | 10.0 | 82.5 |
|  | 40 | 1 | 2.5 | 2.5 | 85.0 |
|  | 41 | 2 | 5.0 | 5.0 | 90.0 |
|  | 43 | 3 | 7.5 | 7.5 | 97.5 |
|  | 45 | 1 | 2.5 | 2.5 | 100.0 |
|  | Total | 40 | 100.0 | 100.0 |  |
