# Supplementary material for: Exploring anxiety awareness during academic science examinations
Source: PLoS One. 2021 Dec 15;16(12):e0261167. doi: 10.1371/journal.pone.0261167 (PMC8673629; doi:10.1371/journal.pone.0261167)
Supplement: S3 Table — (DOCX) [file pone.0261167.s003.docx]

| **Descriptives** | | | | |
| --- | --- | --- | --- | --- |
|  | | | Statistic | Std. Error |
| SUM | Mean | | 34.25 | 1.126 |
|  | 95% Confidence Interval for Mean | Lower Bound | 31.97 |  |
|  |  | Upper Bound | 36.53 |  |
|  | 5% Trimmed Mean | | 34.72 |  |
|  | Median | | 37.00 |  |
|  | Variance | | 50.705 |  |
|  | Std. Deviation | | 7.121 |  |
|  | Minimum | | 15 |  |
|  | Maximum | | 45 |  |
|  | Range | | 30 |  |
|  | Interquartile Range | | 11 |  |
|  | Skewness | | -.920 | .374 |
|  | Kurtosis | | .320 | .733 |
| SUMPOST | Mean | | 30.13 | 1.238 |
|  | 95% Confidence Interval for Mean | Lower Bound | 27.62 |  |
|  |  | Upper Bound | 32.63 |  |
|  | 5% Trimmed Mean | | 30.39 |  |
|  | Median | | 30.50 |  |
|  | Variance | | 61.292 |  |
|  | Std. Deviation | | 7.829 |  |
|  | Minimum | | 14 |  |
|  | Maximum | | 41 |  |
|  | Range | | 27 |  |
|  | Interquartile Range | | 14 |  |
|  | Skewness | | -.417 | .374 |
|  | Kurtosis | | -.791 | .733 |
