# Supplementary material for: Exploring anxiety awareness during academic science examinations
Source: PLoS One. 2021 Dec 15;16(12):e0261167. doi: 10.1371/journal.pone.0261167 (PMC8673629; doi:10.1371/journal.pone.0261167)
Supplement: S4 Table — (DOCX) [file pone.0261167.s004.docx]

| **Tests of Normality** | | | | | | |
| --- | --- | --- | --- | --- | --- | --- |
|  | Kolmogorov-Smirnov^a^ | | | Shapiro-Wilk | | |
|  | Statistic | df | Sig. | Statistic | df | Sig. |
| SUM | .247 | 40 | .000 | .899 | 40 | .002 |
| SUMPOST | .149 | 40 | .026 | .939 | 40 | .033 |
| a. Lilliefors Significance Correction | | | | | | |
