# Supplementary material for: Exploring anxiety awareness during academic science examinations
Source: PLoS One. 2021 Dec 15;16(12):e0261167. doi: 10.1371/journal.pone.0261167 (PMC8673629; doi:10.1371/journal.pone.0261167)
Supplement: S5 Table — (DOCX) [file pone.0261167.s005.docx]

| **Ranks** | | | | |
| --- | --- | --- | --- | --- |
|  | | N | Mean Rank | Sum of Ranks |
| SUMPOST - SUM | Negative Ranks | 28^a^ | 20.48 | 573.50 |
|  | Positive Ranks | 11^b^ | 18.77 | 206.50 |
|  | Ties | 1^c^ |  |  |
|  | Total | 40 |  |  |
| a. SUMPOST < SUM | | | | |
| b. SUMPOST > SUM | | | | |
| c. SUMPOST = SUM | | | | |
