# Supplementary material for: Exploring anxiety awareness during academic science examinations
Source: PLoS One. 2021 Dec 15;16(12):e0261167. doi: 10.1371/journal.pone.0261167 (PMC8673629; doi:10.1371/journal.pone.0261167)
Supplement: S6 Table — (DOCX) [file pone.0261167.s006.docx]

| **Test Statistics^a^** | |
| --- | --- |
|  | SUMPOST - SUM |
| Z | -2.564^b^ |
| Asymp. Sig. (2-tailed) | .010 |
| a. Wilcoxon Signed Ranks Test | |
| b. Based on positive ranks. | |
