# Supplementary material for: Exploring anxiety awareness during academic science examinations
Source: PLoS One. 2021 Dec 15;16(12):e0261167. doi: 10.1371/journal.pone.0261167 (PMC8673629; doi:10.1371/journal.pone.0261167)
Supplement: S7 Table — (DOCX) [file pone.0261167.s007.docx]

| **SUMSF** | | | | | |
| --- | --- | --- | --- | --- | --- |
|  | | Frequency | Percent | Valid Percent | Cumulative Percent |
| Valid | 22 | 2 | 5.0 | 5.0 | 5.0 |
|  | 23 | 3 | 7.5 | 7.5 | 12.5 |
|  | 24 | 3 | 7.5 | 7.5 | 20.0 |
|  | 25 | 2 | 5.0 | 5.0 | 25.0 |
|  | 26 | 4 | 10.0 | 10.0 | 35.0 |
|  | 27 | 5 | 12.5 | 12.5 | 47.5 |
|  | 28 | 2 | 5.0 | 5.0 | 52.5 |
|  | 29 | 5 | 12.5 | 12.5 | 65.0 |
|  | 30 | 6 | 15.0 | 15.0 | 80.0 |
|  | 31 | 2 | 5.0 | 5.0 | 85.0 |
|  | 32 | 3 | 7.5 | 7.5 | 92.5 |
|  | 34 | 3 | 7.5 | 7.5 | 100.0 |
|  | Total | 40 | 100.0 | 100.0 |  |
