# Supplementary material for: Exploring anxiety awareness during academic science examinations
Source: PLoS One. 2021 Dec 15;16(12):e0261167. doi: 10.1371/journal.pone.0261167 (PMC8673629; doi:10.1371/journal.pone.0261167)
Supplement: S8 Table — (DOCX) [file pone.0261167.s008.docx]

| **SUMSFPOST** | | | | | |
| --- | --- | --- | --- | --- | --- |
|  | | Frequency | Percent | Valid Percent | Cumulative Percent |
| Valid | 23 | 1 | 2.5 | 2.5 | 2.5 |
|  | 25 | 2 | 5.0 | 5.0 | 7.5 |
|  | 26 | 1 | 2.5 | 2.5 | 10.0 |
|  | 27 | 2 | 5.0 | 5.0 | 15.0 |
|  | 28 | 5 | 12.5 | 12.5 | 27.5 |
|  | 29 | 11 | 27.5 | 27.5 | 55.0 |
|  | 30 | 7 | 17.5 | 17.5 | 72.5 |
|  | 31 | 5 | 12.5 | 12.5 | 85.0 |
|  | 32 | 1 | 2.5 | 2.5 | 87.5 |
|  | 33 | 4 | 10.0 | 10.0 | 97.5 |
|  | 37 | 1 | 2.5 | 2.5 | 100.0 |
|  | Total | 40 | 100.0 | 100.0 |  |
