# Supplementary material for: Exploring anxiety awareness during academic science examinations
Source: PLoS One. 2021 Dec 15;16(12):e0261167. doi: 10.1371/journal.pone.0261167 (PMC8673629; doi:10.1371/journal.pone.0261167)
Supplement: S9 Table — (DOCX) [file pone.0261167.s009.docx]

| **Tests of Normality** | | | | | | |
| --- | --- | --- | --- | --- | --- | --- |
|  | Kolmogorov-Smirnov^a^ | | | Shapiro-Wilk | | |
|  | Statistic | df | Sig. | Statistic | df | Sig. |
| SUMSF | ,107 | 40 | ,200^*^ | ,966 | 40 | ,260 |
| SUMSFPOST | ,154 | 40 | ,018 | ,949 | 40 | ,069 |
| *. This is a lower bound of the true significance. | | | | | | |
| a. Lilliefors Significance Correction | | | | | | |
