# Supplementary material for: Exploring anxiety awareness during academic science examinations
Source: PLoS One. 2021 Dec 15;16(12):e0261167. doi: 10.1371/journal.pone.0261167 (PMC8673629; doi:10.1371/journal.pone.0261167)
Supplement: S10 Table — (DOCX) [file pone.0261167.s010.docx]

| **Paired Samples Correlations** | | | | |
| --- | --- | --- | --- | --- |
|  | | N | Correlation | Sig. |
| Pair 1 | SUMSF & SUMSFPOST | 40 | ,633 | ,000 |
