# Supplementary material for: Exploring anxiety awareness during academic science examinations
Source: PLoS One. 2021 Dec 15;16(12):e0261167. doi: 10.1371/journal.pone.0261167 (PMC8673629; doi:10.1371/journal.pone.0261167)
Supplement: S11 Table — (DOCX) [file pone.0261167.s011.docx]

| **Paired Samples Test** | | | | | | | | | |
| --- | --- | --- | --- | --- | --- | --- | --- | --- | --- |
|  | | Paired Differences | | | | | t | df | Sig. (2-tailed) |
|  |  | Mean | Std. Deviation | Std. Error Mean | 95% Confidence Interval of the Difference | |  |  |  |
|  |  |  |  |  | Lower | Upper |  |  |  |
| Pair 1 | SUMSF - SUMSFPOST | -1.575 | 2.620 | .414 | -2.413 | -.737 | -3.802 | 39 | .000 |
